# Post-COVID-19 health-care utilization: one year after the 2020 first wave in  Brunei Darussalam

**DOI:** 10.5365/wpsar.2023.14.1.949

**Published:** 2023-01-18

**Authors:** Muhammad Syafiq Abdullah, Rosmonaliza Asli, Pui Lin Chong, Babu Ivan Mani, Natalie Raimiza Momin, Noor Affizan Rahman, Chee Fui Chong, Vui Heng Chong

**Affiliations:** a National Isolation Centre, Ministry of Health, Tutong, Brunei Darussalam.; b Department of Medicine, Raja Isteri Pengiran Anak Saleha Hospital, Bandar Seri Begawan, Brunei Darussalam.; c Pengiran Anak Puteri Rashidah Sa’adatul Bolkiah Institute of Health Sciences, Universiti Brunei Darussalam, Bandar Seri Begawan, Brunei Darussalam.

## Abstract

**Objective:**

Patients who recover from coronavirus disease (COVID-19) infection are at risk of long-term health disorders and may require prolonged health care. This retrospective observational study assesses the number of health-care visits before and after COVID-19 infection in Brunei Darussalam.

**Methods:**

COVID-19 cases from the first wave with 12 months of follow-up were included. Health-care utilization was defined as health-care visits for consultations or investigations. Post-COVID condition was defined using the World Health Organization definition.

**Results:**

There were 132 cases; 59.1% were male and the mean age was 37.1 years. The mean number of health-care visits 12 months after recovery from COVID-19 (123 cases, 93.2%; mean 5.0 ± 5.2) was significantly higher than the prior 12 months (87 cases, 65.9%, *P* < 0.001; mean 3.2 ± 5.7, *P* < 0.001). There was no significant difference when scheduled COVID-19 visits were excluded (3.6 ± 4.9, *P* = 0.149). All 22 cases with moderate to critical disease recovered without additional health-care visits apart from planned post-COVID-19 visits. Six patients had symptoms of post-COVID condition, but none met the criteria for diagnosis or had alternative diagnoses.

**Discussion:**

There were significantly more health-care visits following recovery from COVID-19. However, this was due to scheduled post-COVID-19 visits as per the national management protocol. This protocol was amended before the second wave to omit post-COVID-19 follow-up, except for complicated cases or cases with no documented radiological resolution of COVID-19 pneumonia. This will reduce unnecessary health-care visits and conserve precious resources that were stretched to the limit during the pandemic.

The coronavirus disease (COVID-19) pandemic continues to have significant negative impacts on health-care services worldwide as a result of the diversion of resources to mitigate the impact of the disease, (*1*, *2*) which will have immediate and long-term consequences. Patients affected by COVID-19 are at risk of both medical and psychological long-term health issues. As COVID-19 is predominantly a respiratory illness, long-term respiratory problems are expected. (*3*, *4*) However, a range of adverse outcomes of COVID-19 have also been observed involving the immune system (e.g. Guillain-Barré syndrome and paediatric inflammatory multisystem syndrome), cardiovascular system (e.g. cardiomyopathy and coagulopathy), neurological system (e.g. sensory dysfunction and stroke), cutaneous and digestive manifestations as well as mental health issues. (*4*)

Patients with mild disease from COVID-19 infection who then experienced long-term symptoms (*5*, *6*) are also of concern. This constellation of non-specific symptoms has been referred to as long COVID, chronic COVID syndrome or post-COVID condition, (*5*, *7*) with varying definitions between countries and organizations. The World Health Organization (WHO) defines post-COVID condition as a condition occurring usually 3 months from the onset of COVID-19 with symptoms that last for at least 2 months that cannot be explained by an alternative diagnosis. (*7*) The United States Centers for Disease Control and Prevention (CDC) defines it as a wide range of new, returning or ongoing health problems for 4 or more weeks after COVID-19. (*8*) Common symptoms include fatigue, shortness of breath and cognitive dysfunction that generally impact everyday functioning. (*7*) Symptoms may begin after initial recovery from COVID-19, or may persist from the initial COVID-19 illness, and can fluctuate or relapse over time. The CDC characterized post-COVID conditions into three subtypes: new or ongoing symptoms; multiorgan effects of COVID-19 (i.e. multisystem inflammatory syndrome); and effects of COVID-19 or hospitalization. (*8*) Reported risk factors for chronic sequelae of COVID-19 include disease severity, older age, sex, ethnicity, comorbidities especially pre-existing respiratory disease, and higher body mass index. (*5*, *9*) Female patients have been associated with a higher likelihood of developing mental and psychological long-term sequelae. (*9*, *10*)

To date, few studies have looked at health-care utilization after recovery from COVID-19. (*11*-*15*) One study reported that 10.3% of COVID-19 patients would require re-admission to hospital and an all-cause mortality of 7.9% after recovery from COVID-19, with the majority of deaths occurring within the first 30 days after the index admission. (*12*) The assessment of the burden on the health-care system post-COVID-19 infection from earlier waves can assist with health-care utilization planning. This study of COVID-19 patients from the first wave in Brunei Darussalam aims to: (1) compare health-care utilization of COVID-19 patients 12 months before and 12 months after their infection; (2) assess if severity of disease, underlying psychiatric disorders and need for counselling during hospitalization affected health-care utilization; and (3) assess the prevalence and characteristics of patients diagnosed with post-COVID condition.

## Methods

### Study design

This was a retrospective observational study of cases who recovered from COVID-19 during the first wave (from  9 March 2020 to 6 August 2021) in Brunei Darussalam. All COVID-19 cases in Brunei Darussalam diagnosed during the first wave were admitted to the National Isolation Centre (NIC) for isolation and treatment. All COVID-19 cases from the first wave who were alive 12 months after their COVID-19 recovery and had resided in Brunei Darussalam 12 months before and 12 months after their recovery from COVID-19 were eligible for the study.

To document recovery, scheduled post-COVID-19 health-care visits, as defined in the national post-discharge management protocol, included a reverse transcription polymerase chain reaction (RT–PCR) test on day 11 post-discharge to document viral clearance, and follow-up appointments with cases who had COVID-19 pneumonia as documented on chest radiographs or other unresolved issues directly related to COVID-19 (e.g. thrombocytopenia or unresolved symptoms) at discharge.

### Data collection

Data were retrieved from the database maintained by the NIC management team that had been established at the start of the COVID-19 outbreak. Data collected included age, sex, ethnicity, comorbidities, date of positive RT–PCR test, symptoms at presentation, severity of illness at presentation and daily progress, outcomes and discharge date. Data on health-care utilization during the 12 months before and 12 months after COVID-19 diagnosis were retrieved from the Brunei Darussalam Health and Management System, a national electronic health-care system that links all government health institutions (hospitals and peripheral clinics). Established in 2011, this system captures all patients’ health-care encounters.

Five categories of disease were defined: (i) asymptomatic; (ii) mild (symptomatic without evidence of pneumonia on chest imaging); (iii) moderate (clinical or imaging evidence of pneumonia); (iv) severe (required oxygen supplementation); and (v) critical (respiratory failure requiring mechanical ventilation with or without other organ failure). These were grouped into two categories: asymptomatic/mild and moderate to critical.

### Data analysis

Analyses were conducted using IBM® SPSS version 26.0. Mean, standard deviation and range were calculated for continuous variables and frequency and percentage for categorical variables. The number of health-care visits 12 months before and 12 months after COVID-19 infection were compared. The Mann–Whitney U test was used to test the difference between the mean number of health-care visits for non-parametric continuous variables and the χ^2^ test was used for categorical variables. A *P*-value of < 0.05 was taken as significant.

## Results

### Study population

Of the 340 COVID-19 cases from the first wave, 205 had not resided in Brunei Darussalam 12 months before and 12 months after their recovery from COVID-19 and three had died, leaving 132 cases eligible for the study.

The mean age of the study population was 37.1 ± 17.2 years with more males (59.1%) than females. The ethnic breakdown was consistent with the national distribution. A total of 39 patients (29.5%) had underlying comorbidities, the most common being hypertension and dyslipidaemia (**Table 1**). Nearly half (46.3%) were overweight or obese. Symptoms were reported by 69.7% of cases at admission with the most common being cough (39.0%), fever (26.5%) and rhinorrhoea (23.5%). The majority of cases (83.3%; *n* = 110) had asymptomatic/mild disease and 16.7% (*n* = 22) had moderate to critical disease (**Table 1**). Four cases were admitted to the intensive care unit with two needing mechanical ventilation. The mean length of hospitalization was 20.2 ± 8.7 days.

**Table 1:** Characteristics of COVID-19 cases by mean number of health-care visits 12 months before and  12 months after COVID-19 illness during the first wave,^a^ Brunei Darussalam (*n* = 132)

Characteristic	*n*(%)	Health-care visits 12 months before COVID-19 (mean ± SD)	Overall health-care visits 12 months after COVID-19 (mean ± SD)	*P* ^b^	Non-COVID-19 health-care visits 12 months after COVID-19 (mean ± SD)	*P* ^c^
Sex						
Female	54 (40.9)	3.7 ± 6.1	5.3 ± 5.9	< 0.001	3.9 ± 5.5	0.204
Male	78 (59.1)	3.2 ± 5.2	4.7 ± 4.7	< 0.001	3.3 ± 4.5	0.400
Nationality						
Malay	107 (81.1)	3.7 ± 6.1	5.3 ± 5.6	< 0.001	3.8 ± 5.2	0.340
Chinese	5 (3.8)	1.0 ± 0.7	2.2 ± 1.9	0.310	1.0 ± 1.7	0.548
Other	20 (15.2)	1.7 ± 2.9	4.1 ± 3.1	0.003	2.9 ± 3.0	0.081
Age group (years)						
< 30	48 (36.4)	3.1 ± 6.4	3.7 ± 5.8	0.039	2.6 ± 5.8	0.589
30–50	47 (35.6)	2.8 ± 5.7	5.0 ± 5.3	< 0.001	3.4 ± 4.8	0.160
> 50	37 (28.0)	4.1 ± 4.5	6.7 ± 3.8	0.001	5.0 ± 3.3	0.089
Comorbidities						
Yes	39 (29.5)	5.2 ± 6.8	7.1 ± 5.7	0.014	5.5 ± 5.4	0.294
No	93 (70.5)	2.5 ± 4.9	4.1 ± 4.7	< 0.001	2.7 ± 4.4	0.202
Diabetes						
Yes	9 (6.8)	9.0 ± 10.5	9.1 ± 8.8	0.666	8.1 ± 8.6	0.931
No	123 (93.2)	2.8 ± 4.9	4.7 ± 4.8	< 0.001	3.2 ± 4.4	0.137
Hypertension						
Yes	22 (16.7)	6.4 ± 7.3	8.0 ± 6.4	0.134	6.6 ± 6.1	0.502
No	110 (83.3)	2.6 ± 5.1	4.4 ± 4.8	< 0.001	2.9 ± 4.4	0.147
Dyslipidaemia						
Yes	20 (15.2)	5.9 ± 7.8	6.7 ± 3.9	0.063	5.5 ± 3.7	0.289
No	112 (84.5)	2.8 ± 5.1	4.7 ± 5.4	< 0.001	3.2 ± 5.0	0.229
Ischaemic heart disease						
Yes	5 (3.8)	4.2 ± 3.8	4.4 ± 4.4	1.000	3.8 ± 4.3	0.841
No	127 (96.2)	3.2 ± 5.7	5.0 ± 5.3	< 0.001	3.6 ± 4.9	0.128
Respiratory disease						
Yes	7 (5.3)	2.0 ± 2.8	6.4 ± 5.2	0.073	4.6 ± 4.5	0.535
No	125 (94.7)	3.3 ± 5.8	4.9 ± 5.2	< 0.001	3.5 ± 4.9	0.192
Reported symptoms at admission						
Yes	92 (69.7)	3.5 ± 6.4	5.3 ± 5.3	< 0.001	3.7 ± 4.9	0.131
No	40 (30.3)	2.7 ± 3.4	4.4 ± 5.1	0.020	3.2 ± 4.9	0.741
Abnormal chest radiography^d^						
Yes	22 (17.9)	3.8 ± 4.9	9.6 ± 6.1	0.001	6.5 ± 6.1	0.019
No	101 (82.1)	3.1 ± 5.9	4.2 ± 4.6	< 0.001	3.1 ± 4.6	0.387
Disease severity						
Asymptomatic/mild	110 (83.3)	3.0 ± 5.7	4.0 ± 4.5	< 0.001	2.9 ± 4.6	0.471
Moderate to critical	22 (16.7)	4.7 ± 5.5	10.0 ± 5.8	0.001	7.0 ± 6.0	0.062

^a^ The first wave lasted from 9 March 2020 to 6 August 2021.

^b^ Comparison between health-care visits 12 months before COVID-19 and overall health-care visits 12 months after COVID-19.

^c^ Comparison between health-care visits 12 months before COVID-19 and non-COVID-19 health-care visits 12 months after COVID-19.

^d^ Nine cases (children) did not undergo chest radiography.

### Health-care utilization

**Fig. 1:**
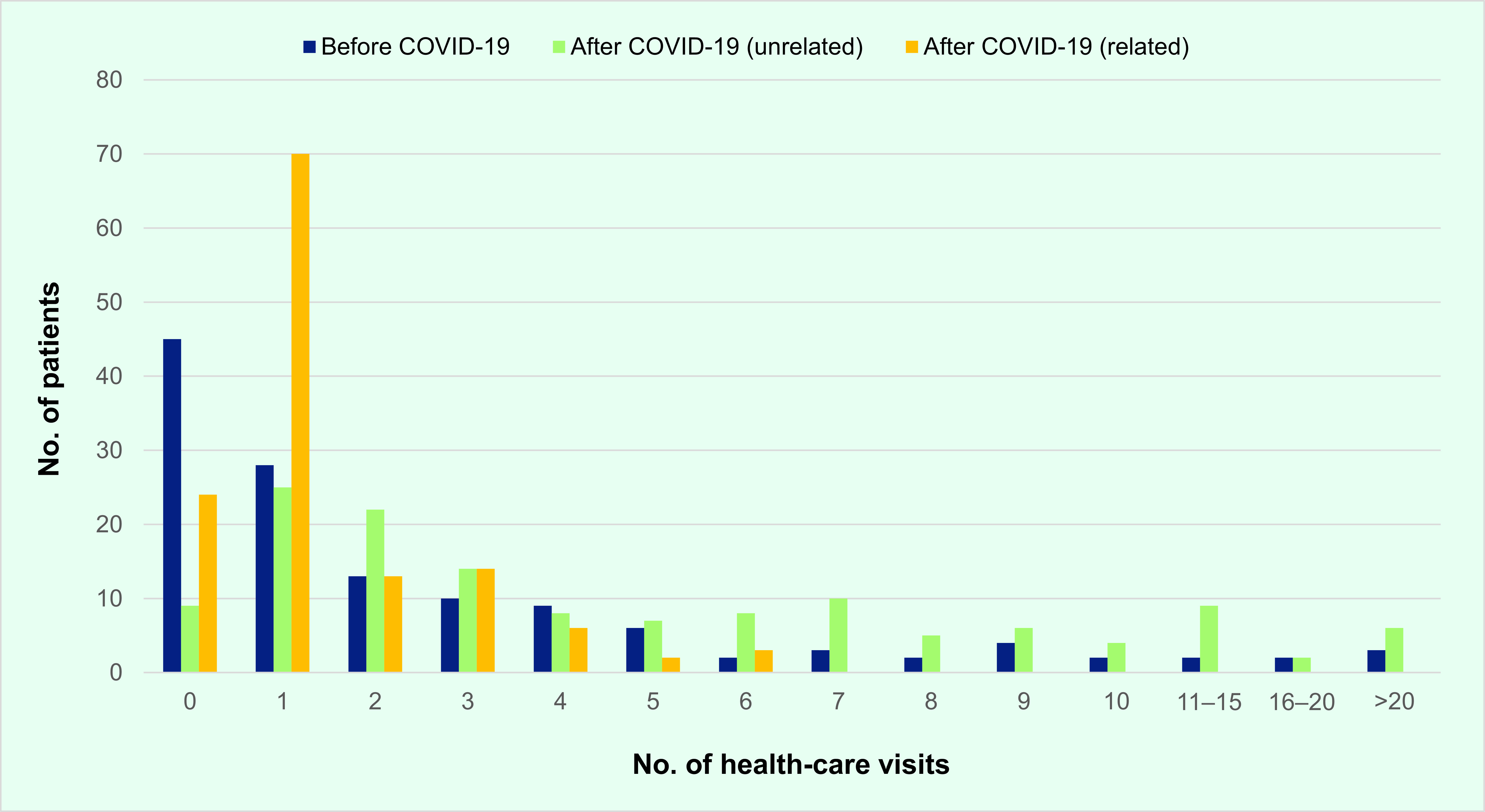
Distribution of COVID-19 cases during the first wave^a^ by number of health-care visits 12 months before illness, and number of health-care visits unrelated and related to COVID-19 in the 12 months after recovery, Brunei Darussalam (N = 132)

Most cases (64.4%) visited health-care facilities 12 months before and 12 months after recovering from COVID-19 (**Table 2**). **Fig. 1** shows the breakdown in the number of health-care visits before and after COVID-19 (unrelated and related to COVID-19). This shows scheduled COVID-19-related visits ranging from one to six visits, most with one visit, mainly for post-discharge testing for severe acute respiratory syndrome coronavirus 2 (SARS-CoV-2) to document viral clearance following our management protocol at the time.

[**insert** Figure 1]

**Table 2:** Proportion of cases during the first wave^a^ with health-care visits 12 months before and 12 months after COVID-19 illness, Brunei Darussalam (*n* = 132)

Health-care visits before/after COVID-19 illness	*n*(%)
No/No	7 (5.3)
No/Yes	38 (28.8)
Yes/No	2 (1.5)
Yes/Yes	85 (64.4)

^a^ The first wave lasted from 9 March 2020 to 6 August 2021.

Overall, there were significantly more health-care visits (*n* = 660, mean 5.0 ± 5.2 visits) in the 12 months after COVID-19 compared to the 12 months before (*n* = 431, mean 3.2 ± 5.7; *P* < 0.001). There was a significant increase in the mean number of visits observed between each characteristic assessed except for Chinese ethnicity (**Table 1**). Cases with comorbidities (diabetes mellitus, hypertension, dyslipidaemia, ischaemic heart disease and respiratory disorders) had more health-care visits compared to those without comorbidities. However, there was no significant increase in health-care visits post-COVID-19.

There were 190 scheduled post-COVID-19 visits, with a mean of 1.4 ± 1.3 per case (range 1–6). When scheduled post-COVID-19 visits were excluded, there was no significant difference between the mean number of health-care visits pre- and post-COVID-19 (*n* = 470, mean 3.6 ± 4.9; *P* = 0.149). Similarly, when scheduled COVID-19 visits were excluded, there was no significant difference between the mean number of health-care visits for each characteristic assessed pre- and post-COVID-19, except for patients with abnormal chest radiography (*P* = 0.019).

Among non-COVID-19 health-care visits, there were 11 for COVID-19 vaccinations: five partial (one dose) and three complete (two doses).

### Patients with moderate to severe COVID-19 disease

There were 22 (16.7%) cases with COVID-19 pneumonia (moderate to critical disease) including two who required mechanical ventilation. Eleven had radiological resolutions documented at discharge and 11 had complete resolutions documented at follow-up. All were cleared of any residual respiratory issues. None had any further health-care visits for respiratory or other problems related to COVID-19 other than their scheduled post-COVID-19 visits.

### Psychiatric encounters

During hospitalizations for COVID-19, six patients required counselling or psychiatric treatment (**Table 3**), four of whom were diagnosed with underlying mild psychiatric disorders during admission but did not have prior encounters with public or mental health-care services. One case was referred due to concern about prolonged hospitalization and because their family members had recovered much earlier. Four patients were given treatment. Post-discharge, four had follow-up appointments, of whom two were already known to the service and two were new. Both cases 5 and 6 had improved when they were reviewed. One was seen once before missing her scheduled follow-up appointment, and the other patient continued routine follow-up (**Table 3**).

**Table 3:** Encounters with psychiatric counselling services 12 months before COVID-19 infection, during hospitalization and 12 months after recovery during the first wave,^a^ Brunei Darussalam (*n* = 132)

-	12 months before COVID-19	During hospitalization for COVID-19	12 months after COVID-19
Encounters, *n*(%)	4 (2.9)	6 (4.4)	4 (2.9)
Case no.: Disorder	1: Psychotic depression 2: Learning disability 3: Autism spectrum disorder (paediatric) 4: Autism spectrum disorder (paediatric)	2: Learning disability (risk of impulsivity/aggression) 5: Anxiety and panic attacks 6: Anxiety and panic attacks 7: Anxiety disorder (reactive anxiety and insomnia) 8: Attention deficit hyperactivity disorder 9: Concern of staff	1: Psychotic depression 2: Learning disability (lost to follow-up) 5: Anxiety and panic attacks 6: Anxiety and panic attacks

^a^ The first wave lasted from 9 March 2020 to 6 August 2021.

### Post-COVID condition

Six patients had some symptoms of post-COVID condition but none met the criteria for diagnosis. Four of these patients had hospital encounters within 60 days and two after 8 months following their initial COVID-19 infection (**Table 4**). Three patients had pre-existing psychiatric disorders, which were exacerbated by COVID-19 illness in two of these patients. The third patient had transient forgetfulness which the patient described as brain fog. Psychometric evaluations for this patient were normal.

**Table 4:** Cases with symptoms of post-COVID condition during the first wave,^a^ Brunei Darussalam (*n* = 6)

Case no.	Sex/age (years)	Disease severity	Length of hospitalization (days)	Pre-existing condition	Symptoms	Outcomes	Last consult	Days between discharge and first health-care visit
5	Female/23	Mild	14	Yes: Anxiety	Anxiety, palpitation, insomnia, nightmares	Resolved	Discharged	20
6	Female/23	Mild	17	Yes: Anxiety and panic	Anxiety attacks	Resolved	Discharged	18
8	Male/39	Mild	23	Yes: Attention deficit hyperactivity disorder	Forgetfulness/unable to find words, unable to concentrate	Resolved	Discharged	255
10	Male/43	Moderate	20	No	Localized chest pain and itchy rash	Resolved	Discharged	36
11	Male/62	Moderate	33	No	Palpitation	Diagnosed with supraventricular tachycardia	Cardiology follow-up	54
12	Female/19	Mild	35	No	Atypical chest pain, cramps, choking sensation	Bulimia	Still on follow-up	284

^a^ The first wave lasted from 9 March 2020 to 6 August 2021.

One case developed palpitations 54 days after discharge and investigations revealed idiopathic supraventricular tachycardia. Coronary angiography assessment before diagnosis of COVID-19 was normal. Another case developed non-specific symptoms which resolved, although they were later diagnosed with bulimia, and the final case had transient localized musculoskeletal chest pain (Tietze syndrome) (**Table 4**).

## Discussion

Our study showed a significantly higher mean number of health-care visits among recovered COVID-19 cases from the first wave in Brunei Darussalam 12 months after recovery compared with the 12 months before infection. However, this increase in health-care visits was mainly due to scheduled post-COVID-19 health-care visits as per the national management protocol at the time. Although some cases had symptoms of post-COVID condition, none fulfilled the WHO criteria for diagnosis (*7*) or they had alternate diagnoses, and their symptoms were self-limiting. None of the cases with COVID-19 pneumonia had long-term respiratory effects during the 12 months after recovering from COVID-19.

Post-COVID condition is a well recognized disorder, (*7*, *8*) with varying definitions regarding symptoms and duration. Although there were cases with some symptoms of post-COVID condition, all had alternative diagnoses to account for their symptoms, either due to exacerbations of pre-existing conditions, chest musculoskeletal pain similar to Tietze syndrome or cardiac arrhythmias that were unrelated to COVID-19. Some of our cases did meet the definition of other diagnostic criteria, including the CDC criteria. (*5*, *8*) Fortunately, most cases recovered without further consultations or treatment, indicating that post-COVID-19 symptoms were mild and self-limiting. However, it remains to be seen if post-COVID condition will be a significant problem in our setting with a larger number of patients affected by COVID-19 in subsequent waves.

Our findings differ from other studies reported in the literature. A meta-analysis of 91 studies showed a prevalence of hospital readmissions during the first 30 days, 90 days and 1 year post-discharge of 8.97%, 9.79% and 10.34%, respectively. (*12*) Most cases of hospital readmissions occurred within 30 days after discharge. (*12*) A study from Switzerland of 385 patients with COVID-19, 81 of whom required hospitalization during initial illness, reported that at 6–8 months after illness, 26% (*n* = 111) had not fully recovered, 40% (*n* = 170) reported at least one visit to the general practitioner and 10% (*n* = 81) of those hospitalized were re-hospitalized. (*11*) Individuals who had not fully recovered or had fatigue, dyspnoea or depression were more likely to have further health-care contacts. However, a third of individuals (37/111) who had not fully recovered did not seek further care. (*11*) This indicated that despite residual symptoms persisting, they may not have been significant enough to require health-care visits. The difference between our findings and those of other studies may be due to the small total number of patients affected by COVID-19 in Brunei Darussalam during the first wave, including those categorized as severe. However, it is possible that the difference is due to factors such as vulnerability or susceptibility to post-COVID-19 illness, and is influenced by social, cultural and religious factors. (*16*, *17*) Other factors may also be at play and will require further study.

There are many reasons why patients may have physiological or psychological issues after recovery from COVID-19. (*4*-*9*) Apart from patients with COVID-19 pneumonia and a case of transient thrombocytopenia, none of the cases from this study had any other symptoms. As previously reported, cases in this cohort with moderate to critical COVID-19 all had abnormal chest radiography. (*18*) All cases were reviewed by the respiratory department, cleared of any long-term pulmonary issues and eventually discharged to their primary care doctors. None had further health-care visits for pulmonary issues. In the first wave, only chest radiography was used for imaging as computed tomography, which is superior in detecting respiratory changes due to COVID-19, was not available. (*19*) If it had been available and used, this would have likely resulted in more unnecessary scheduled post-COVID-19 visits. One study has reported persistent air exchange dysfunction after recovering from COVID-19. (*20*) It is uncertain if symptoms will become apparent after a much longer period and, therefore, longer follow-up studies are required.

The mental well-being of COVID-19 patients is likely to be impacted either directly due to their COVID-19 infection or as a psychological impact of implemented restrictive measures. (*4*, *10*) Six of our patients needed counselling during their hospitalizations. Common indications for counselling were anxiety-related issues that were exacerbated by COVID-19 illness. This was not surprising, given that at the time COVID-19 was a novel viral illness without effective treatment. Furthermore, our management protocol required all COVID-19 cases to be admitted for isolation in single isolation rooms or warded with strangers for a minimum duration of 14 days. (*21*) Movement was also restricted to the wards or rooms. This was further compounded by frequent medical investigations (blood draws, radiological imaging and nasopharyngeal swabbing). All these can incur anxiety and fear in addition to stressors brought on by the COVID-19 illness itself. However, this did not translate to additional health-care visits.

This study of the first wave of COVID-19 in Brunei Darussalam showed that most patients recovered without further issues and significant post-COVID conditions were uncommon. COVID-19 remains a novel infectious disease, especially with new SARS-CoV-2 variants of concern appearing. However, the knowledge gained has resulted in a better understanding of COVID-19, as reflected in changes to our national management protocols. After the peak of the first wave in 2020, post-discharge testing was omitted as it was shown that the number of cases re-testing positive after discharge was not insignificant. (*22*-*24*) Longer follow-up for non-resolving symptoms or laboratory monitoring also stopped and instead cases were directed to their primary care clinics. The current management protocols require follow-up for patients with unresolved chest radiography findings with moderate to severe COVID-19 or for those who had a complicated illness.

There are several limitations that need to be considered when interpreting our findings. Using encounters from government health-care institutions based on the electronic record system excluded encounters with private clinics. However, the demographics of our study patients are consistent with patients whose health-care needs are usually met by the public sector. Furthermore, encounters with the private sector are likely to be minor and considered non-significant, as in Brunei Darussalam specialty services are only available from public health-care institutions. The sample size was small, and further studies with larger cohorts would be useful and should be considered. Prior to the second wave, there were only 340 patients with COVID-19 in Brunei Darussalam and, of these, the majority were excluded as they were expatriates who had just entered Brunei Darussalam or did not have 12 months of follow-up. Despite these limitations, the study population was representative of the situation in Brunei Darussalam. The small number of cases may account for differences between our study and other literature with higher re-admission rates. Studies on post-COVID condition would likely capture more cases by survey rather than electronic records, as patients with milder conditions may self-manage and not present at a health-care facility. However, our study is unique in that our findings are representative of the whole country as all COVID-19 cases were admitted to a single designated centre.

In conclusion, our study showed that there were significantly more health-care visits 12 months after recovery from COVID-19 compared with the 12 months before infection. However, most post-COVID-19 health-care utilization was due to scheduled COVID-19 health-care visits. Post-COVID condition was not officially diagnosed, and related symptoms were mild and self-limiting. However, our sample size was small and this is a limitation that needs to be taken into account. Further studies are required with a larger sample size. The larger cohort of patients affected by the second wave in Brunei Darussalam would be ideal for further study.
